# Overcoming challenges in designing and implementing a phase II randomized controlled trial using a presurgical model to test a dietary intervention in prostate cancer

**DOI:** 10.1177/1740774508091676

**Published:** 2008

**Authors:** Wendy Demark-Wahnefried, Stephen L George, Boyd R Switzer, Denise C Snyder, John F Madden, Thomas J Polascik, Mack T Ruffin, Robin T Vollmer

**Affiliations:** ^a^Division of Cancer Prevention & Control/Department of Behavioral Science, MD Anderson Cancer Center, Houston, TX USA, ^b^Department of Biostatistics, Duke University Medical Center (DUMC), Durham, NC, USA, ^c^Department of Nutrition, University of North Carolina, Chapel Hill, NC, USA, ^d^Duke School of Nursing, Durham, NC, USA, ^e^Department of Pathology, DUMC, Durham, NC, USA, ^f^Department of Surgery, DUMC, Durham, NC, USA, ^g^Department of Family Medicine and University of Michigan Community Clinical Oncology Program Research Base, University of Michigan, Ann Arbor, MI, USA, ^h^Durham Veterans Administration Medical Center, Durham, NC, USA

## Abstract

**Background:**

The time between the diagnosis of cancer and a planned definitive surgical procedure offers a strong and direct approach for assessing the impact of interventions (including lifestyle interventions) on the biology of the target tissue and the tumor. Despite the many strengths of presurgical models, there are practical issues and challenges that arise when using this approach.

**Purpose/Methods:**

We recently completed an NIH-funded phase II trial that utilized a presurgical model in testing the comparative effects of flaxseed supplementation and/or dietary fat restriction on the biology and biomarkers associated with prostatic carcinoma. Herein, we report the rationale for our original design, discuss modifications in strategy, and relay experiences in implementing this trial related to the following topics: (1) subject accrual; (2) subject retention; (3) intervention delivery; and (4) retrieval and completion rates regarding the collection of paraffin-embedded and fresh frozen prostate tissue, blood, urine, ejaculate, anthropometric measures and survey data.

**Results:**

This trial achieved its accrual target, i.e., a racially-representative (70% white, 30% minority) sample of 161 participants, low rates of attrition (7%); and collection rates that exceeded 90% for almost all biospecimens and survey data. While the experience gained from pilot studies was invaluable in designing this trial, the complexity introduced by the collection of several biospecimens, inclusion of a team of pathologists (to provide validated readings), and shifts in practice patterns related to prostatectomy, made it necessary to revise our protocol; lessons from our experiences are offered within this article.

**Conclusions:**

While our experience specifically relates to the implementation of a presurgical model-based trial in prostate cancer aimed at testing flaxseed-supplemented and fat-restricted diets, many of the lessons learned have broad application to trials that utilize a presurgical model or dietary modification within various cancer populations.

## Introduction

Lifestyle factors are postulated to play a key role in the development and progression of prostate cancer (PC) [1,2]. Diet is presumed to play an especially strong role, yet the evidence base for undertaking dietary change with the intent of cancer prevention or control is lacking. To date, much of what is known about diet and PC comes from epidemiologic investigations that are limited in their ability to show cause and effect, *in vitro* studies or interventions in animal models which may or may not have relevance to the disease course in humans, or small clinical pilot studies that lack adequate control or that rely on indirect endpoints of disease (e.g., prostate specific antigen [PSA])[2]. Hence, we look to large-scale randomized controlled trials (RCT), such as the Selenium and Vitamin E Cancer Prevention Trial (SELECT) [3], to provide us with convincing evidence that dietary factors do indeed make a difference; however, these trials are both time- and resource-intensive [4,5]. The presurgical model has been proposed as an efficient means of assessing the impact of various interventions on PC [6]; this model also can be applied to studies of dietary factors. The purpose of this article is to describe the original design, shifts in strategy and the lessons learned while implementing an NIH-funded (R01 CA85740) phase II RCT that utilized the presurgical model to compare the effects of dietary fat restriction and/or flaxseed supplementation on PC and its associated biomarkers.

## Background

The presurgical or preoperative model has been proposed to evaluate a wide spectrum of interventions in various site-specific cancers [6]. In PC, it has experienced limited use, though recently a small study was reported that assessed changes in serum proteomic patterns with pre-surgical vitamin E and selenium supplements as a subproject of the SELECT trial [7]. In that 2 × 2 placebo-controlled study, 48 PC patients were randomly assigned to receive vitamin E and/or selenium during the 3–6 weeks prior to prostatectomy, and the proteomic patterns of their sera collected at baseline were compared to those of sera after treatment. That protocol differs from typical presurgical designs where survey data and most biological samples (blood, urine, semen, etc.) are collected at baseline and just prior to surgery, but where excised tumor also is assessed for relevant endpoints. This more complete use of the presurgical model was used in our phase II RCT.

Our exploration of the potential benefits of dietary manipulation began with an incident case of a patient with high grade prostatic intraepithelial neoplasia (HGPIN) who demonstrated remarkable reductions in PSA and notable cellular atrophy upon repeat biopsy after adhering to a flaxseed-supplemented, low-fat diet. Given that flaxseed is an exceptionally rich source of both dietary lignans and omega-3 fatty acids, dietary factors that have been found to influence androgen and COX-2 metabolism (the effects on the COX-2 pathway may be further enhanced by a low-fat diet which may accelerate the conversion of alpha-linolenic acid [ALA : 18 : 3n-3] to longer chained and more physiologically active omega-3 fatty acids, eicosapentanoic [EPA : 20 : 5n-3] and docosahexanoic [DHA : 22 : 5n-3] acids)[8–14], we followed-up this observation with three studies: (1) an *in vitro* experiment that exposed three major PC cell lines (LNCaP, DU-145, and PC-3) to flaxseed-derived mammalian lignans [15]; (2) an animal feeding experiment that tested a 5% flaxseed-supplemented diet against an isocaloric-controlled diet in transgenic mice genetically programmed to develop PC [16]; and (3) a pilot feasibility study of a low-fat, flaxseed-supplemented diet in men found to have HGPIN or atypia [17]. In all of these studies, flaxseed derived lignans, flaxseed itself or the flaxseed diet with concomitant dietary fat restriction were found to significantly hinder prostatic growth (via decreased proliferation and increased apoptosis in both malignant and benign tissue), and/or reduce tumor burden.

While our original plan was to test flaxseed supplementation and dietary fat restriction in men with HGPIN [18], the experience gained from our feasibility study suggested that this would be an unfruitful strategy for several reasons: (1) there is poor inter-rater concordance regarding the classification of HGPIN between pathologists [19]; (2) the diagnosis of HGPIN is relatively rare without co-occurring PC [20], making recruitment difficult; and (3) HGPIN is often absent upon rebiopsy [17]. The possibility of using a presurgical model [6] in PC patients was intriguing but we were concerned about the effect of a dietary modification on the biology of the prostate if only implemented for a few weeks. While theoretically the presurgical model could be undertaken for a much longer length of time, i.e., months or even years, this is not a feasible option for PC patients, who are anxious about their disease and unlikely to postpone their treatment. Thus, we conducted a pilot study to test the feasibility of the presurgical model by enrolling 25 men scheduled for prostatectomy, who followed a flaxseed-supplemented, low-fat diet [21]. Accrual to the study was much more rapid than that of the previous HGPIN study [17], and study subjects were on the diet for an average of 34 days (range 21–77 days) during which time they experienced significant decreases in serum testosterone, cholesterol and free androgen index. A nonsignificant (*p* = 0.10) reduction in PSA also was observed among the subset of patients with Gleason sum 6 or less disease. Most compelling, however, was that compared to historic controls matched on age, race, PSA at diagnosis and biopsy Gleason sum, men on the diet had tumors with significantly lower proliferation rates and higher rates of apoptosis. Thus, the presurgical model appeared to offer a feasible means to test our dietary intervention and we used this body of research to justify a phase II trial to test the comparative effects of flaxseed-supplementation and/or dietary fat restriction on PC.

## Methods and design considerations

### Design

Given that our pilot studies combined flaxseed supplementation with dietary fat restriction, there was a need to disentangle potential effects of each. One possible design, often used in similar settings, involves comparing the effects of treatment interventions on the change from baseline. In our setting, this would involve the difference between the result from the diagnostic biopsy and the result from the surgical specimen (i.e., using the ‘patient as his own control’). However, unless a ‘no treatment’ control group is included, the substantial heterogeneity within the target (prostate gland in our case) and the very limited sampling of the target in the original biopsy could lead to biased results. Thus, we employed a 2 × 2 factorial design [22] with a no treatment control group and outcomes measured on each subject at the end of the treatment period. The treatments were defined through the presence or absence of the two factors, flaxseed supplementation and dietary fat restriction, defining the following four treatments: (1) control; (2) flaxseed-supplemented diet (FS); (3) low-fat diet (LF); and (4) flaxseed-supplemented, low-fat diet (FSLF). It also would have been desirable to utilize placebo controls, but this was not feasible either for flaxseed supplementation or for the low-fat diet. Unlike micronutrient supplementation trials that rely on small dosages and which lend themselves to a placebo-controlled design, the 30 g. daily dose flaxseed tested was substantial and nonamenable to a suitable placebo, since other fibers, even cellulose, have been shown to affect hormonal levels [23]. Also, for obvious reasons, it was not possible to blind low-fat diet conditions. Therefore, in this single-blinded study, we attempted to reduce potential contamination of the control group through the following strategies: (1) emphasizing the importance of control group participation among men assigned to this arm; (2) offering men results of their dietary analysis and guidance to improve their diets once they had recovered from their surgery; (3) administering a detailed food frequency questionnaire at both baseline and follow-up from which data could be used to control for unplanned dietary change; and (4) assessing biomarkers associated with a low fat diet (serum lipids) and flaxseed consumption (urinary lignan metabolites), which also could be used to assess the potential for contamination and confounding.

### Endpoints

We originally considered PSA velocity as the primary endpoint in the study but PSA is influenced by a myriad of factors and may not necessarily reflect the status of the cancer. Thus tumor proliferation rate, as assessed by murine hybridoma (MIB-1) staining of the prostatectomy specimens was selected as our primary endpoint, since it was used successfully in our previous studies. The MIB-1 marker (also known as k:-67) also has validated use in nutrition intervention trials [24], and has been endorsed by the Prostate Cancer Chemoprevention Trial Consensus Panel as an accurate and reproducible measure [25]. While apoptosis, as assessed by terminal deoxynucleotidyl transferase (*T*dT)-mediated d*U*TP- *n*ick *e*nd-*l*abeling (TUNEL), also was considered, the fact that we did not observe adequate variation within our pilot studies caused us to relegate this to a secondary endpoint. The specific aims of the study were to determine differences between the four study arms with respect to the following: (1) tumor proliferation as assessed by MIB-1 staining of prostatectomy specimens (primary aim), as well as rates of apoptosis using TUNEL (secondary aim); (2) changes in biomarkers that reflect or are associated with PC growth and/or are influenced by a low-fat diet or flaxseed supplementation, such as serum PSA, total testosterone, sex hormone binding globulin (SHBG), insulin-like growth factor (IGF-1), IGF binding protein-3 (IGFBP-3), c-reactive protein (CRP), and serum lipids (secondary aim) [21,26–29]; (3) changes in nutritional biomarkers (i.e., levels of lignans in the urine and seminal fluid, and fatty acid profiles of circulating erythrocytes and prostatic tissue); and (4) to explore associations between dietary change, change in dietary biomarkers, change in hormonal intermediates, and change in study endpoints (secondary aim).

### Power and Sample Size Calculations

We tested the null hypothesis *H*_0_ : μ_0_ = μ_1_ against the two-sided alternative *H*_1_ : μ_0_ ≠ μ_1_ where μ_ι_ (*i* = 0 for ‘control’, *i* = 1 for FS or LF) was the mean logarithm of the MIB-1 proliferation index and used two primary tests, one for FS and one for LF. Our preliminary studies suggested that the combination of FS and LF resulted in log proliferation rates that were on average 33% lower than the log proliferation rates observed among control subjects. This translated into an observed effect size of approximately 0.56. Given some hesitation in relying solely upon an effect size from a study which employed historic controls, we also banked on the findings of Hamalainen *et al*. who also found a similar effect size in a fiber-supplement trial aimed at testosterone reduction [30]. To achieve a statistical power of 0.80 (two-sided test, α = 0.05) at an effect size of 0.50, a total of 128 participants (32 per cell) was needed. An additional eight participants per cell was added to cover losses due to drop-out (4% attrition in the pilot study) and to allow for a slight negative interaction [31]. Thus, the total target sample size was 160 patients (40 in each cell).

### Eligibility

Inclusion and exclusion eligibility criteria are listed in Table 1; most exclusionary criteria were instituted to reduce potential confounding.

**Table 1:** Inclusion and exclusion criteria

Inclusion criteria	Exclusion criteria
• Men with pathologically confirmed prostate cancer who elected prostatectomy as their primary initial treatment	• Men who had received hormonal or neo-adjuvant chemotherapy
• Men who were scheduled for prostatectomy at one of the participating study sites and who were at least 21 days from surgery	• Men currently adhering to a low fat diet (the 16-item NCI Percent Energy from Fat Screener was programmed into Microsoft Excel and used in real-time to screen-out men whose diets were ≤30% of energy from fat) [27].
• Mentally-competent, English-speaking and -writing men with telephone access (interventions and evaluative surveys were based in English and relied on written materials and telephone counseling)	• Men currently consuming flaxseed
	• Men who would be taking antibiotics within the study period (antibiotic-use inhibits the intestinal conversion of flaxseed lignans to mammalian-based lignans [40].
	• Men taking dietary supplements that were newly started or who planned to initiate use during the study period. At the origin of the study, the definition for “newly started dietary supplement” encompassed “*any* dietary supplement initiated within 6-months,” this definition was revised soon after launching into the field when it became clear that we were excluding a very high proportion of men; our revised criterion excluded those who started dietary supplements [not including standard multi-vitamin preparations] within the past 3 months.

### Accrual

PC patients were recruited from the urology clinics at Duke University Medical Center (DUMC), the Durham Veteran's Administration Medical Center (DVAMC), and five sites associated with the University of Michigan Cooperative Community Oncology Program (CCOP) Research Base. Written consent was obtained using two different methods depending on the study site IRB requirements and convenience for the patient. In most cases, patients were initially contacted by telephone, the study was explained and interest in the study was elicited. Patients who were not interested were thanked for their time and then asked to volunteer their reasons for disinterest; this information was de-identified and recorded. Patients who expressed an interest were screened for eligibility, and reasons for ineligibility also were tracked in a deidentified database. To help defray costs associated with study participation and reduce potential barriers to accrual, men were offered a monetary incentive of $100 if they completed the study.

Men who were both eligible and interested in participating were either asked to report for a pre-baseline appointment (where written consent was obtained and men were given surveys to complete at home, as well as containers [coolers and ice packs] and instructions to collect a 24-h urine sample and an ejaculate sample or were express mailed a consent form, surveys and directions and containers for specimen collection. In either case, written consent was obtained prior to the collection of specimens. Written consent was obtained for the study itself and additional consent was obtained for permission to utilize any unused biospecimens, as well as to assess the whole blood sample for DNA once definitive genes for PC were identified. All men were asked to report for both the baseline and follow-up appointments after a 12-h fast; follow-up appointments were scheduled within 3-days of surgery. To reduce the variation associated with diurnal fluctuation with some of the endpoints (e.g., testosterone), baseline and follow-up appointments were scheduled in the morning. Additionally, because PSA may be influenced by prostatic manipulation, baseline appointments were scheduled at least 14 days post-biopsy and at least 3 days post-digital rectal exam [32].

### Baseline appointment and measures

Blood was drawn via venipuncture and either kept as whole blood or configured into sera and plasma. Samples were aliquotted and stored at −70°C until completion of the study, whereupon they were batch-analyzed via immunochemiluminometric assay (LabCorp, Inc., Burlington, NC) for PSA, total testosterone, SHBG, total and low density cholesterol, CRP, IGF-1, and IGFBP-3. Erythrocyte membranes also were isolated, stored and batch-analyzed upon study completion via capillary gas chromatography (GC) to discern fatty acid composition [33].

Start and stop times for 24-h urine collections were recorded, and samples (which arrived chilled in coolers) were measured for volume, mixed, and aliquotted. One aliquot was immediately analyzed for creatinine using kinetic methods (DUMC Clinical Laboratories and LabCorp, Inc, Burlington, NC) to assure a 24 h collection and to use as a benchmark for expressing lignan excretion (a marker of dietary adherence to the flaxseed-supplemented regimen); urinary lignans were quantified using high performance liquid chromatography (HPLC) [34,35]. Similar methods were used to chronicle ejaculate collection and to analyze the lignan content of seminal fluid [34,35]. As with urine, converted plant-lignans are expressed in the seminal fluid, and findings of Morton *et al*. suggest a reduced risk of PC not only among men, who consume higher amounts of dietary lignans, but who also express higher concentrations of lignans in their seminal fluid [11,34].

Given increasing evidence that body weight affects the progression of prostatic carcinoma and may confound study findings [36], men were measured for height using a wall-mounted stadiometer and measured for weight using a routinely-calibrated digital scale; body mass index (BMI) was derived using the formula kg/m^2^. Given a need to control for other dietary factors (lycopene, vitamin E, etc.) and physical activity, men also were asked to complete the Diet History Food Frequency Questionnaire (modified to include regionally-consumed foods, such as okra, hominy, grits, and various organ meats)[37,38], and the Aerobics Center Longitudinal Study Physical Activity Questionnaire [39]. Information regarding various socio-demographic factors, medical history, prescribed and over-the-counter medications and supplements also was recorded.

### Randomization

After all baseline data and biospecimens were collected (the exception being ejaculate which several men either refused or were unable to provide), men were block randomized on prognostic factors of race (black vs. non-black) and biopsy Gleason sum (<7 vs. 7+) [1], to one of four treatment arms: (1) control; (2) flaxseed-supplemented diet; (3) low-fat diet; and (4) flaxseed-supplemented, low-fat diet. The randomization process was designed and carried out by the biostatistics group in the Duke Comprehensive Cancer Center. While the treatment arms differed with regard to dietary regimen, men in all arms were contacted weekly by the study staff to maintain contact (this was especially important since surgeries often were rescheduled) and to assess and reinforce adherence, and to answer any diet-related questions. Additionally, wives of participants (or partners responsible for food procurement or preparation) were encouraged to attend the baseline appointment to enhance the quality of dietary instruction should men be randomized to one of the modified diet arms.

#### Control arm

Men in this arm were asked to continue their usual diet and not to make any changes in their dietary intake.

#### Flaxseed-supplemented (FS) arm

Men assigned to this arm were provided with ample ground flaxseed to last until their date of surgery. To reduce the variability in nutrient composition that could occur between crops, the flaxseed used for this study was obtained from ENRECO, Inc. (Manitowoc, WI) in one lot (150 kg), and was analyzed for nutrient content at two time points during the study period. Given its propensity for rancidity [40], the flaxseed was stored in whole grain form under cold storage, and ground and packaged in daily dose (30 g) sealed opaque packets as needed (note that flaxseed must be ground, or the hull split, in order to enhance the absorption of its omega-3 fatty acids and lignans). Starter kits with stepped doses were provided, such that for days 1–3, packets contained only 10 g of ground flaxseed, for days 4–6 packets contained 20 g of flaxseed, and by day 7 the full 30 g dose was provided. This stepped dose approach was considered necessary given the considerable fiber load that flaxseed imposes on the gut (∼9 g/30 g dose), and the propensity for gastrointestinal discomfort if initiated in full-dose fashion. Men receiving flaxseed also were instructed to drink at least 64 oz. of fluids/day to reduce any potential risk of colonic impaction or dehydration resulting from the increased fiber load [41], and to keep their flaxseed packets under refrigeration (to reduce spoilage). In addition, participants in this arm were provided with logs to record their daily intakes of flaxseed to the nearest quarter of a packet, and to return any unused packets at the time of their follow-up appointment. These procedures were adapted from pill counts which have been found to provide a valid measure of adherence in pharmacologic trials (including fiber supplement trials)[41,42].

#### Low-fat arm (LF)

Men randomized to this arm were instructed by registered dietitians on a diet containing ≤20% of total energy from dietary fat. Fat gram ‘budgets’ were individually calculated for each participant using the following formula: ideal body weight (lb) × 15 × 0.2 kcal from fat/9 kcal/gram. Men were provided with fat gram counters and asked to record all foods consumed along with the corresponding number of fat grams, and to tally the number of fat grams consumed each day. Participants also received written and verbal instruction on meal planning, food preparation, shopping, and dining.

#### Flaxseed-supplemented, low-fat diet (FSLF) arm

Men in this arm received instruction and supplies for both of the diet regimens described above.

### Follow-up appointment and measures

Within three days of surgery, and most often on the morning of surgery, participants reported for the follow-up appointment. At this time, participants were asked to report any changes in health status and/or new medications, and all measures (except height) performed during the baseline appointment were repeated. Additionally, all participants were asked to complete CALGB toxicity index scales for potentially relevant side effects, i.e., nausea, vomiting, diarrhea, impotence/libido, and allergy [43]. Men assigned to diet modified arms also were asked to rate their adherence to the FS (average number of days/week flaxseed was consumed and the average amount) and/or LF diets (average number of days/week they adhered to fat gram budgets). These adherence measures served as yet another measure of dietary intake, in addition to the DHQ, record logs, and biomarkers (i.e., urinary lignans).

Upon prostatectomy, fresh, frozen tissue was retrieved from two defined regions in the central and peripheral zones of the prostate using a 3 mm punch biopsy instrument. These defined regions for biopsy were selected based on the following rationale:(1) samples would be extracted from the interior prostate and therefore would not interfere with surgical margin assessment; and (2) zones slated for harvest had a lower probability of including tumor and therefore less likely to be needed for patients’ clinical work-ups. Each of the two tissue samples was flash frozen in liquid nitrogen, placed in individual cryovials and stored at −70°C until completion of the study. It should noted that the prostatectomy tissue harvested at any of the CCOP sites remained on-site until the full clinical work-up for each patient was complete, just in case the extracted sample was needed for clinical assessment. Once released, the sample was shipped on dry ice to the CCOP coordinating center and then onto DUMC. At completion of the study, prostate tissue homogenates were batch-analyzed via GC to discern fatty acid composition using methods described previously.

The primary study pathologist (RV) reviewed clinical pathology reports and slides for each case and determined the blocks that should be cut and prepared for determination of proliferation (primary endpoint) and apoptosis in both tumor and benign tissue. Proliferation rates were assessed using antibody from the MIB-1 clone at a dilution of 1 : 200 (Biocare, Walnut Creek, CA) [24]. Prepared slides were independently reviewed by both the primary study pathologist (RV) and the secondary study pathologist (JFM) who were blinded with regard to study arm, using the following method: (1) at low magnification a random starting point in the tumor was chosen; and (2) then at high magnification, an average of 547 sequentially-encountered tumor cell nuclei were evaluated for MIB-1 positivity (range 110–2550). The result was reported as the ratio of positive nuclei divided by the total number evaluated. Values obtained from the two pathologists were then averaged and mean values used in statistical calculations. The degree of apoptosis was measured using TUNEL [44]. Labeled nuclei were assessed by the two study pathologists using the methods described above and under fluorescence microscopy. Slides were given a rank score of 0 (lowest apoptotic index), 1 or 2 (highest apoptotic index), and averaged values were used in statistical analyses.

### Statistical analysis

A pre-planned interim analysis was pursued (at a nominal significance level of 0.001) after 50% of the subjects had been enrolled. At that time, results indicated that the null hypothesis could not be rejected and accrual continued until the target sample size was achieved. Data collection for the trial is now complete and data analyses are underway. Statistical regression techniques were used to analyze the effect of variables such as race, age, biopsy Gleason sum, weight loss, exercise, dietary fat and lignan intakes, and physiologic markers associated with dietary modification (i.e., concentrations of lignan in the prostatic fluid, prostatic tissue, and the urine and lipid composition of the prostate) on hormonal intermediates (i.e., androgen levels, IGF-1 and IGFBP-3), and ultimately on study endpoints (i.e., rates of proliferation and apoptosis and PSA change). Secondary analyses explored whether the results differed among men who were upgraded upon surgery (i.e., those whose surgical Gleason sums exceeded those at biopsy, which may be a particular issue among men who have Gleason sums of 6 or less at biopsy and then transition to higher scores upon surgery) [45]. Thus, biopsy may have misclassified men who would have otherwise been stratified differently.

### Conduct of the study/overcoming barriers to completion

**Figure 1:**
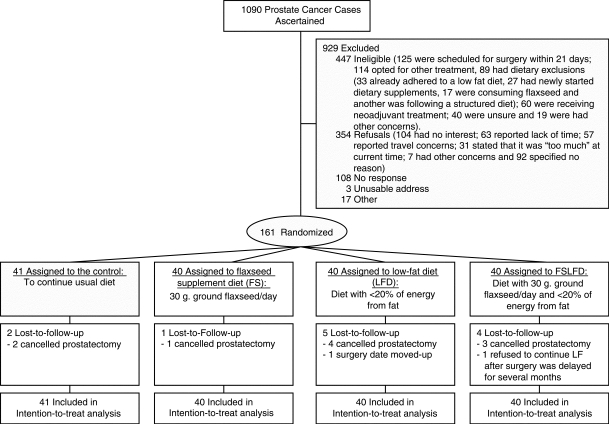
Study flow diagram

This study accrued patients from July 5, 2002 to April 17, 2006, and the recruitment index (number of days to accrue one analyzable study subject) was 8.9 days [46]. This differed from original projections which assumed a recruitment index of ∼6.8 days and proposed a 3-year window to achieve targeted accrual. Furthermore, at the time this study was conceived (2000), we assumed that accrual could be achieved solely with the clinic loads available at DUMC and DVAMC, relying on surgical case loads that approximated 300 cases/year. Months into the trial, it became apparent that this strategy was flawed due to higher than anticipated numbers of men who opted for treatments other than prostatectomy (a treatment trend that also resulted in a decreased wait-time prior to prostatectomy). Newly armed with knowledge that the proportion of patients eligible for this presurgical trial was significantly lower in this age of expanding treatment options, we forged a collaboration with the University of Michigan-CCOP to expand our recruitment effort. Among all sites a total of 1090 men were actively screened for this trial and 161 were ultimately enrolled (Figure 1). Roughly 48% of actively screened subjects were ineligible with leading reasons for ineligibility being: (1) opting for treatment other than surgery or undecided regarding treatment choice; (2) scheduled for surgery within 21 days; (3) dietary exclusions; and (4) receiving neoadjuvant treatment. A refusal rate of ∼38% also was observed with leading reasons for refusal being: (1) general disinterest or no expressed reason, (2) lack of time; and (3) transportation concerns. Time and travel issues are common reasons for nonparticipation in clinical trials, thus such findings were anticipated. Under other reasons for refusal, a substantive proportion of men voiced discomfort in collecting 24-h urine and ejaculate samples, and although we discontinued the requirement to provide the latter sample (since it was only a secondary endpoint and a noted barrier to accrual) these study procedures may have been off-putting, not only for those who specified the reason for their refusal, but also those who expressed general disinterest with no stated reason.

Table 2 provides demographic characteristics of the study sample. Data suggest the accrual of a diverse sample that was racially representative of the population from which it was ascertained. Given the difficulties reported regarding minority recruitment to clinical trials, it is clear that this trial serves as a success [47]. This achievement may have been due to the use of a monetary incentive and the efforts of the study staff to piggyback both baseline and follow-up appointments onto existing clinic appointments. While the age of study participants was roughly a decade younger than the mean age reported for the onset of PC [48], it must be remembered that this sample was extracted from a population scheduled for surgery and notably younger.

**Table 2:** Characteristics of the study sample

	Total
Age (years)	
Mean (SD)	59.2 (7.3)
Range	36–73
Race – % (*n*)	
White	70% (112)
African American	26% (42)
Other	4% (7)
Education – % (*n*)	
<High School/unknown	9% (15)
High School Grad/GED	20% (32)
Some College/Trade	29% (46)
College Grad/Post-grad	42% (68)
Biopsy Gleason Sum – % (*n*)	
<7	68% (110)
7+	32% (51)

Baseline appointments, randomization and weekly contact proceeded smoothly and as planned. As the study progressed, higher percentages of men preferred to be contacted via email than by telephone, and this helped conserve study staff time and resources. Only one adverse event was reported throughout the course of study implementation: one study participant stood-up suddenly, lost consciousness and suffered a nonsevere head injury (but one that required an emergency room visit). This event was not considered diet-related.

A low attrition rate of only 7% was observed, with 10-of-12 drop-outs discontinuing the study because they subsequently decided against surgery. In such cases, we attempted to schedule follow-up appointments to collect biospecimens and surveys that would be useful in secondary analysis; however, this was not always possible since some men refused to travel if no longer scheduled for treatment. Reasons for the two other drop-outs also involved prostatectomy, in one case the participant's surgery was moved-up by three weeks and in another, the subject's surgery was delayed by many months and the participant did not want to adhere to a low-fat diet for an extended period of time. No socio-demographic differences were observed between drop-outs and study completers.

This trial was exceptionally complex with regard to sample and data collection. Success rates regarding specimen retrieval and data collection among the primary study site (DUMC/DVAMC) and CCOP sites are reported in Table 3. Retrieval of paraffin-embedded tissue for MIB-1 analysis (primary endpoint) as well as TUNEL was 100%. The collection of fresh frozen tissue achieved lower retrieval rates of 89–96%, likely owing to the fact that this is a time-sensitive and nonstandard procedure. In cases where tissue was not successfully retrieved, it always involved a breakdown in communications between the study staff and personnel of the pathology laboratory who formalin-fixed the prostatectomy specimens before cores of fresh tissue could be harvested. Collection of blood, urine, anthropometric measures and survey data also was highly successful with rates generally surpassing 90%. Indeed, the primary problem regarding sample retrieval related specifically to the collection of ejaculate, where rates were notably low among the primary study site (58% at baseline/39% at follow-up) and even lower at CCOP sites (24% at baseline/18% at follow-up). While the study protocol originally called for extraction of seminal fluid via prostatic massage, this technique was abandoned for two reasons: (1) poor success in obtaining ample fluid from older men who were likely to comprise our study sample; and (2) concern that this procedure would impose a considerable barrier to recruitment. It is unknown if our poor success rate in obtaining ejaculate could be overcome by other methods or if this is an inherent issue in this population. It is noteworthy that collection rates decreased significantly at follow-up and could have resulted from the fact that many follow-up appointments were scheduled on the day of surgery (when increased anxiety may have interfered with collection).

**Table 3:** Record of successful specimen retrieval or survey/measure completion

	Baseline		Follow-up
	DUMC/DVAMC	CCOP	DUMC/DVAMC	CCOP
Paraffin-embedded tissue			121/121	28/28
Fresh frozen tissue			116/121	25/28
Blood samples				
8.5 cc serum separator tube	132/132	29/29	126/126	28/28
2–5 cc lithium heparin tubes	132/132	29/29	125/126	28/28
3 cc EDTA-treated tube	132/132	29/29	126/126	27/28
24-h urine collection	131/132	29/29	124/126	28/28
Ejaculate sample	77/132	7/29	49/126	5/28
Measured height	132/132	29/29		
Measured weight	132/132	29/29	126/126	28/28
Completed diet history questionnaire	129/132	26/29	123/126	26/28
Completed physical activity survey	132/132	28/29	123/126	28/28
Completed Logs				
fat grams			59/59	15/15
flaxseed			59/59	18/18
CALGB toxicity index			123/126	28/28
Diet self-assessment			91/92	24/28

### Summary

PC trials that employ presurgical models have several strengths and can ultimately measure the impact of interventions directly on the target tissue. While this model provides scientific rigor, it is balanced by several pragmatic obstacles – obstacles that are heightened by the diversification of treatment and the many therapeutic options available to men with this disease. Indeed, we were able to overcome numerous barriers in implementing this phase II dietary trial which employed the presurgical model. We successfully recruited and retained a diverse study sample, effectively delivered the experimental interventions, and retrieved a majority of biospecimens and completed data collection instruments. Our strategies and methods may assist others who contemplate chemoprevention studies that utilize presurgical models not only in PC, but also cancers of the breast, cervix, head and neck, bladder, and colon.
